# Gelation Behaviors and Mechanism of Silk Fibroin According to the Addition of Nitrate Salts

**DOI:** 10.3390/ijms17101697

**Published:** 2016-10-10

**Authors:** Dong Su Im, Min Hee Kim, Young Il Yoon, Won Ho Park

**Affiliations:** Department of Advanced Organic Materials and Textile System Engineering, Chungnam National University, Daejeon 34134, Korea; lds7477@naver.com (D.S.I.); vvvkmhvvv@nate.com (M.H.K.)

**Keywords:** silk fibroin, gelation, nitration, tyrosine

## Abstract

Silk fibroin (SF) is a typical fibrous protein that is secreted by silkworms and spiders. It has been used in a variety of areas, and especially for tissue-engineering scaffolds, due to its sound processability, mechanical properties, biodegradability, and biocompatibility. With respect to gelation, the SF gelation time is long in aqueous solutions, so a novel approach is needed to shorten this time. The solubility of regenerated SF is sound in formic acid (FA), which is a carboxylic acid of the simplest structure. In this study, SF was dissolved in formic acid, and the addition of salts then induced a rapid gelation that accompanied a solution-color change. Based on the gelation behaviors of the SF solution according to different SF and salt concentrations, the gelation mechanism was investigated.

## 1. Introduction

Silk, which is secreted by silkworms, spiders, mites, and pseudo-scorpions, is a generally fibrous protein that has attracted considerable attention due to its inherent optical and outstanding mechanical properties [1,2,3,4]. It has been widely used in high quality textile industries, but has recently played a significant role in the medical materials for surgical sutures and as a wound dressing with respect to membranes [5,6]. It has been known that silk mainly consists of two proteins whereby fibroin is a dominant component (75%), and that it is also hydrophobic [7,8]. Contrary to fibroin, sericin comprises a hydrophilic property with a fibroin encasement for protection. Fibroin is composed of a light chain with 26 kDa and a heavy chain with 350 kDa [9]. The structure has a strong influence on the strength and elasticity of silk. The amino acid composition of silk is made up of glycine, alanine and serine, all of which represent more than 90% of the total content [10]; their short side chains make intermolecular stacking interactions facilitative, and this leads to an antiparallel β-sheet structure of a high crystalline quality [11]. The primary protein structure of fibroin holds the hydrophobic protein structure of the natural block co-polymer [12].

The biocompatibility of fibroin is not only sound, but it also comprises transformable properties for the preparation of an aqueous solution by a variety of methods [13]. Due to its strong benefits, fibroin has been steadily researched in terms of cosmetics and food additives [14], and its application has recently been extended to fields such as artificial blood vessels, wound dressings, and drug delivery on account of its inherent biological properties [15].

The results from the gelation of fibroin include intermediate properties between liquids and solids, a porous structure, and elasticity by three-dimensional crosslinking [16]. A variety of methods have been introduced to gelate fibroin, for example a radical reaction using irradiation and a bridging reaction using chemical covalent bonds or cross-linking agents [17].

For all of their effective performances, such as the time necessary to gelate silk fibroin (SF) solution, a limitation still exists. In the reported SF gelation study, 1 to 50 h gelation times were spent to form the SF gel [18]. The aim of this study is to dramatically reduce the gelation time through the addition of salts. The proposed method induced a rapid gelation of SF solution and the gelation process was confirmed using diverse analysis methods [19,20,21].

## 2. Results and Discussion

### 2.1. Gelation Behavior of Silk Fibroin (SF)/Formic Acid Solution According to the Addition of a Variety of Salts

**Figure 1:**
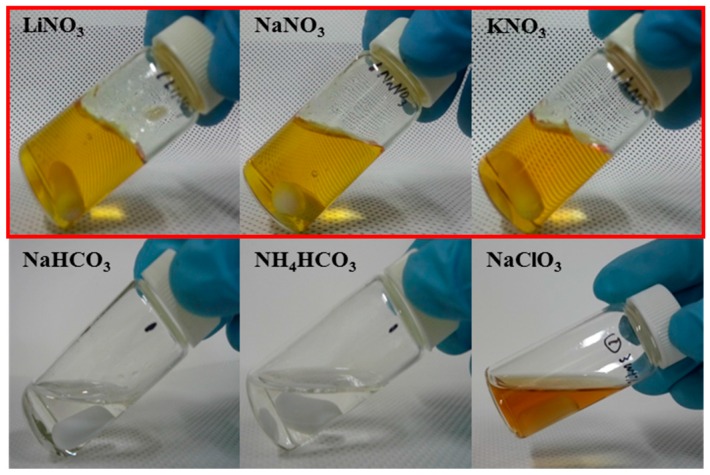
Gelation behaviors of silk fibroin (SF)/formic acid solution according to the addition of salts. The red parts indicate gelated SF solutions according to the addition of nitrate-type salts.

The addition of salts with nitrate in the SF/formic acid solution led to immediate gelation reactions. All of the salt concentrations were 3.5% of the SF weight, and the concentration of the SF/formic acid solution was 5%. Interestingly, a fast reaction rate was shown when nitrate-type salts such as sodium nitrate (NaNO_3_), lithium nitrate (LiNO_3_), and potassium nitrate (KNO_3_) were treated (Figure 1). However, when salts without nitrate such as sodium bicarbonate (NaHCO_3_) and ammonium bicarbonate (NH_4_HCO_3_) were added to the solution, no changes were observed. After the addition of NaClO_3_, a brownish color was observed without gelation. This finding indicates that the gelation reaction of the SF/formic acid solution is derived from the addition of the nitrate salts, and the nitrate salts induced the growth of the solution as yellowish gels. The reaction was completed within a few minutes and yellow gels were obtained.

### 2.2. Effect of SF and NaNO_3_ Concentration on Gelation

**Figure 2:**
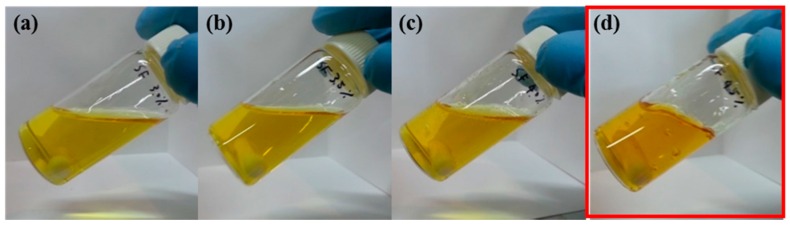
Concentration effect of the SF/formic acid solution on gelation: (**a**) 3% SF; (**b**) 3.5% SF; (**c**) 4% SF; and (**d**) 4.5% SF. The red part indicates a gelated SF solution at a 4.5% SF concentration.

The gelation behavior was observed under varying concentrations of the SF/formic acid solution where NaNO_3_ is a representative nitrate salt. In the 3% to 8% SF/formic acid solution with 3.5% NaNO_3_, gelation occurred above the 4.5% SF concentrations (Figure 2).

**Figure 3:**
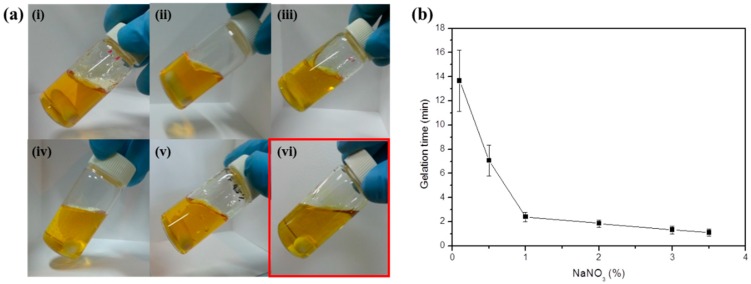
(**a**) Effect of NaNO_3_ concentration on gelation: (**i**) 0.1% NaNO_3_; (**ii**) 0.5% NaNO_3_; (**iii**) 1% NaNO_3_; (**iv**) 3% NaNO_3_; (**v**) 3.5% NaNO_3_ and (**vi**) 4% NaNO_3_; (**b**) Gelation time of SF solution according to NaNO_3_ concentration. The red part indicates a non-gelated SF solution at a 4% NaNO_3_ concentration.

For the gelation of SF, the minimum concentration of the SF/formic acid solution is 4.5%. Also in the 0.1% to 4% NaNO_3_ and 4.5% SF concentrations, gelation occurred below the 3.5% NaNO_3_ concentration. The maximum gelation concentration of NaNO_3_ is 3.5% at a 4.5% SF/formic acid solution (Figure 3a(i–vi)). When 0.1% NaNO_3_ was added to the SF/formic acid solution, the gelation time was 14 min, while the gelation of the 3.5% NaNO_3_ took 1 min. Therefore, the gelation times of the SF/formic acid were decreased under high concentrations of NaNO_3_ due to the increased gelation reaction rate (Figure 3b). Gelation time was measured below 3.5% NaNO_3_ because at 4% NaNO_3_, gelation did not occur. Further experiments were performed at a 5% SF concentration to obtain stable SF gels.

### 2.3. Viscosity Change According to the NaNO_3_ Concentration

**Figure 4:**
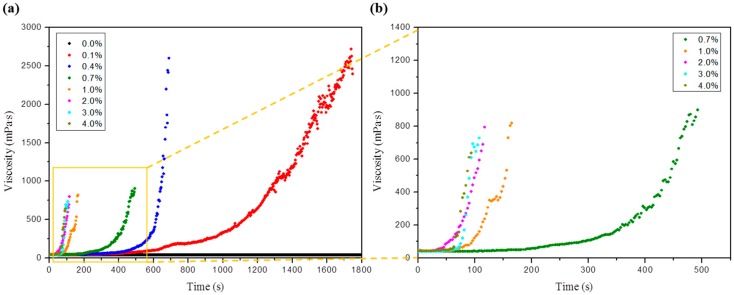
Viscosity change of SF gel depending on concentration of NaNO_3_: (**a**) viscosity change of SF gel from 0% to 4% NaNO_3_ concentrations and (**b**) the expanded viscosity change of SF gel at high NaNO_3_ concentrations (from 0.7% to 4%).

The addition of NaNO_3_ to the SF/formic acid solution induced an increased viscosity regarding the gelation reaction. The viscosities were therefore analyzed according to the addition of diverse NaNO_3_ concentrations (Figure 4). Before the addition of NaNO_3_, the viscosity of the SF/formic acid solution was similar to water, but the high NaNO_3_ concentrations showed a low viscosity during gelation. The concentration of the NaNO_3_ was decreased, the viscosity of the SF gel became higher, and the gel stability was improved.

### 2.4. Compositional Change of SF upon Gelation

#### 2.4.1. Amino Acid Analysis

**Figure 5:**
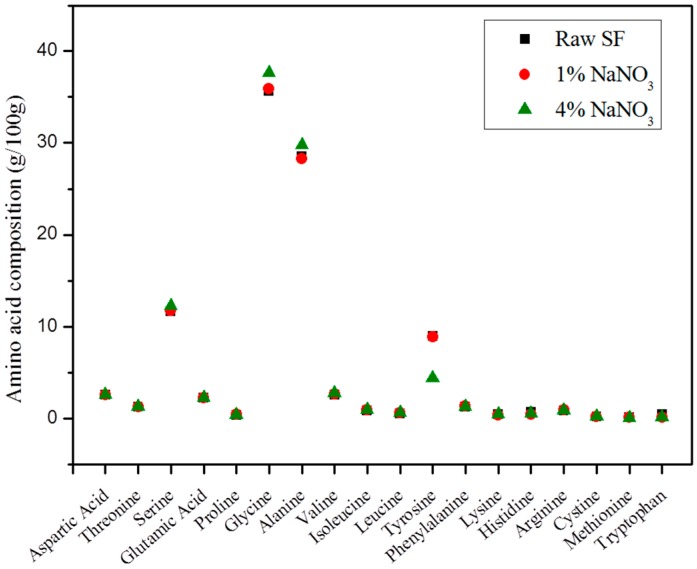
Amino acid composition of SF gel depending on the NaNO_3_ concentration.

**Figure 6:**
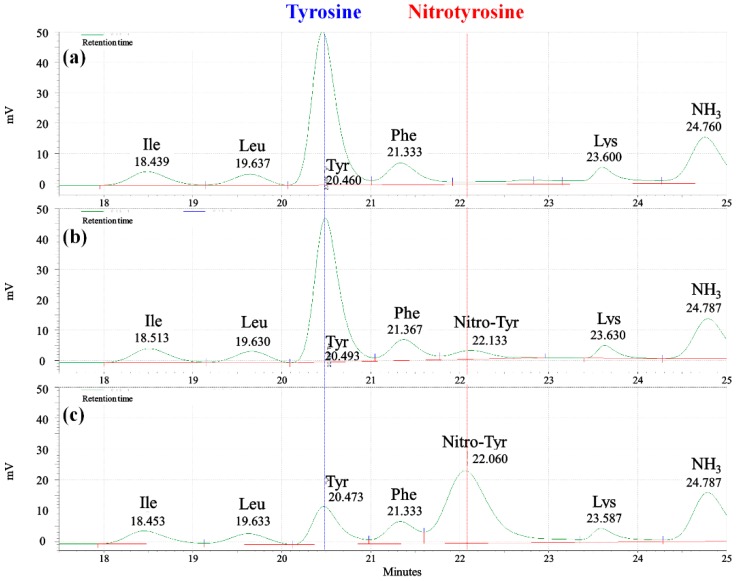
Contents of tyrosine and nitrotyrosine in SF gel depending on the NaNO_3_ concentrations: (**a**) Raw SF; (**b**) 1% NaNO_3_; and (**c**) 4% NaNO_3_.

SF consists of 18 amino acids including Gly, Ala, Ser, and Tyr, and this amino acid composition was investigated according to the SF gelation (Figure 5). The concentration of the SF/formic acid is 5%, and except for tyrosine, content changes were not observed in the amino acids of the SF gel. Tyrosine content decreased when the NaNO_3_ concentration increased and a nitrotyrosine peak appeared (Figure 6). A standard test using 3-nitro-l-tyrosine confirmed that the nitration of the tyrosine modified it to nitrotyrosine. The nitrotyrosine content was increased when the NaNO_3_ concentration was increased.

#### 2.4.2. UV-Vis Spectroscopy

**Figure 7:**
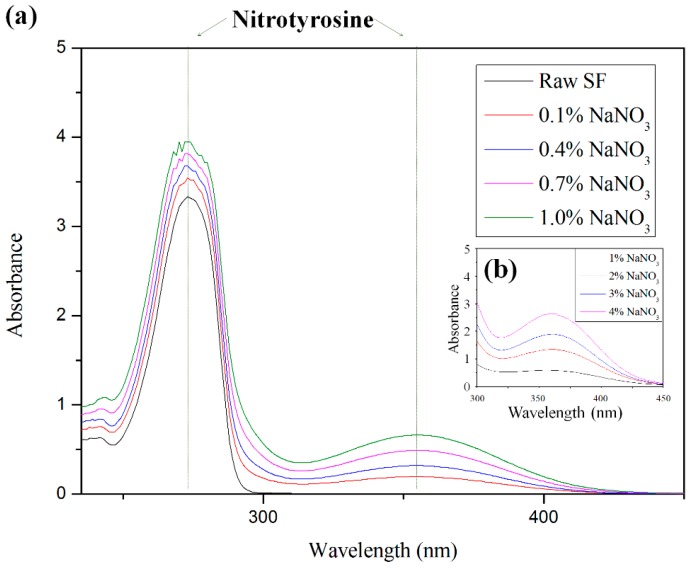
UV-vis spectroscopy results of SF gels depending on the NaNO_3_ concentration: (**a**) 0% to 1% NaNO_3_ and (**b**) 1% to 4% NaNO_3_.

The functional SF groups were investigated upon gelation using a UV-vis spectrophotometer (UV-2450, Shimadzu, Japan) (Figure 7). As the NaNO_3_ concentration increased, typical nitrotyrosine peaks increased at 274 nm and 356 nm [22], indicating that the tyrosine reacted to the NaNO_3_, and that the nitration of the SF modified the tyrosine to nitrotyrosine. This finding is consistent with the results of the amino acid analysis of the SF gel [23].

### 2.5. Fluorescence Spectroscopy

**Figure 8:**
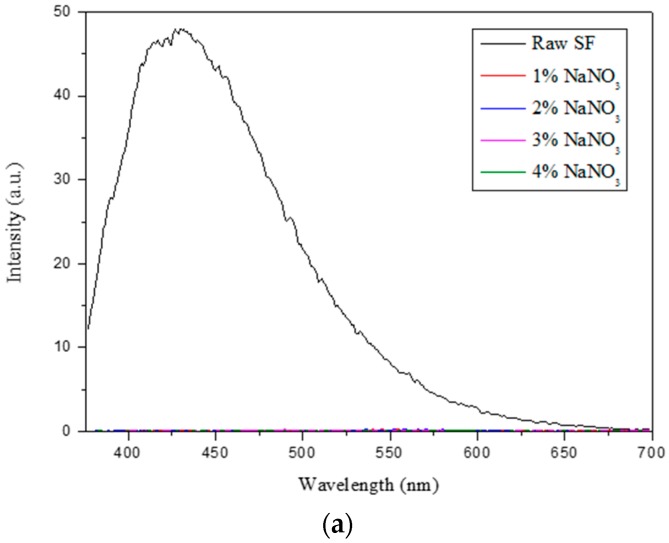

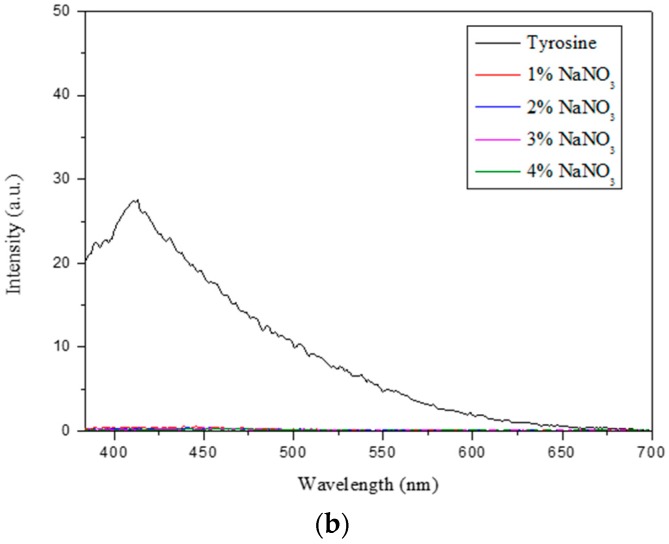
Fluorescence intensity comparison of SF gels: (**a**) 1% to 4% NaNO_3_ and (**b**) with tyrosine/formic acid solution (Excitation wavelength: 365 nm).

The fluorescence intensity change upon gelation was observed through the fluorescence property of the tyrosine in the SF (Figure 8). When gelation occurred in the SF/formic acid solution, the fluorescence intensity of tyrosine at 426 nm (excitation wavelength: 365 nm) was decreased by the increase of the NaNO_3_ concentration. In a control experiment for which the tyrosine/formic acid solution was used, when the NaNO_3_ concentration was treated, the tyrosine fluorescence intensity at 416 nm was decreased in a similar manner as the original solution, indicating that the tyrosine content decreased upon gelation due to the nitration of the tyrosine in the SF [24].

### 2.6. Mechanism of SF Gelation and Nitration

**Figure 9:**
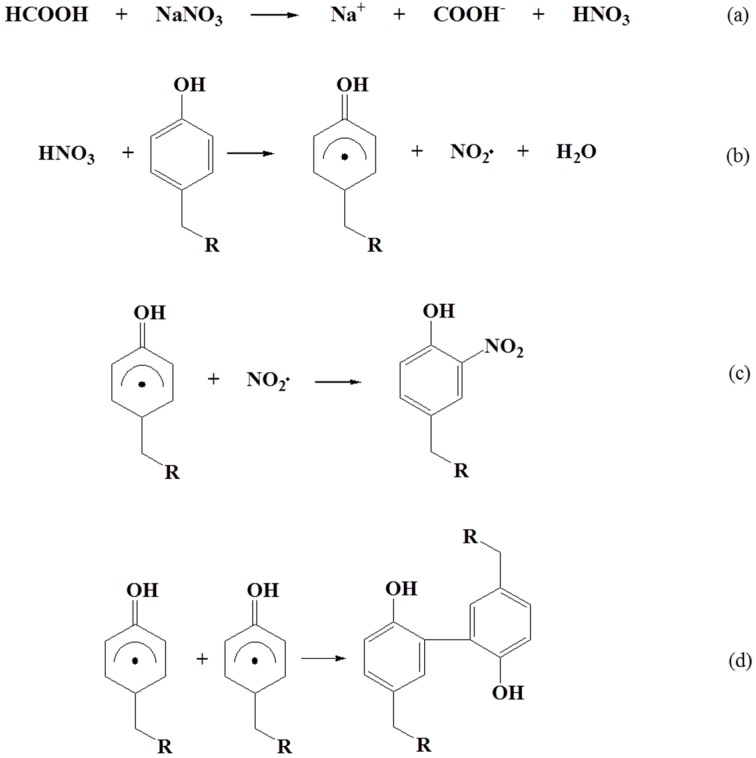
Gelation mechanism of SF/formic acid solution with NaNO_3_. (**a**) addition of NaNO_3_; (**b**) tyrosyl radical formation; (**c**) nitration of tyrosine; (**d**) cross-linking of tyrosine.

The solubility of SF in water is low because of its hydrophobic property, but it was easily dissolved in the formic acid. When NaNO_3_ was dissolved in the SF/formic acid solution, it was dissociated in the formic acid solvent and reacted with the hydrogen cation to form NO_3_^−^ (Figure 9a). The tyrosine reacted with the NO_3_^−^-synthesized tyrosyl radical and NO_2_ radical (NO_2_∙) (Figure 9b). The prepared NO_2_∙ might be formed to nitrotyrosine through a nitration reaction between the NO_2_∙ and tyrosyl radical. Also, the two tyrosyl radical groups probably cross-linked with each other to form a dityrosine structure [25,26]. In addition, this was followed by the induction of the gelation in the SF/formic acid solution (Figure 9c,d). For a confirmation of this mechanism, a further study will be performed.

## 3. Materials and Methods

### 3.1. Materials

The raw silk was reeled off the cocoons of the *Bombyx mori* silkworm. The three types of nitrate salts NaNO_3_, LiNO_3_, and KNO_3_ were used, and these salts were purchased from Samchun (Pyeongtaek, Korea). The three types of non-nitrate salts NaClO_3_, NaHCO_3_, and NH_4_HCO_3_ that were obtained from Sigma–Aldrich (St. Louis, MO, USA) were used, and these salts were used as additives in the SF solution. The formic acid (98%) was purchased from Junsei (Tokyo, Japan). The l-tyrosine and 3-nitro-l-tyrosine were also purchased from Sigma-Aldrich.

### 3.2. Preparation of SF/Formic Acid Solution

Raw-silk fibers were degummed using a 0.5% (*w*/*w*) sodium bicarbonate (NaHCO_3_) solution at 100 °C for 30 min before they were rinsed with warm distilled water [27]. While the degummed silk fibroin was insoluble in formic acid, the regenerated SF was readily soluble in formic acid [28]. The degummed SF was dissolved in a ternary solvent system of calcium chloride/ethanol/water (1:2:8 in molar ratio) at 85 °C for 4 h. After dialysis with a cellulose tubular membrane (molecular cut-off, 12,000) in distilled water for three days, the aqueous SF solution was filtered and freeze-dried to obtain regenerated SF sponges [29,30]. The SF solution was prepared by dissolving the regenerated SF sponges in formic acid for 30 min [28].

### 3.3. Gelation Behavior of SF/Formic Acid Solution According to the Addition of a Variety of Salts

**Figure 10:**
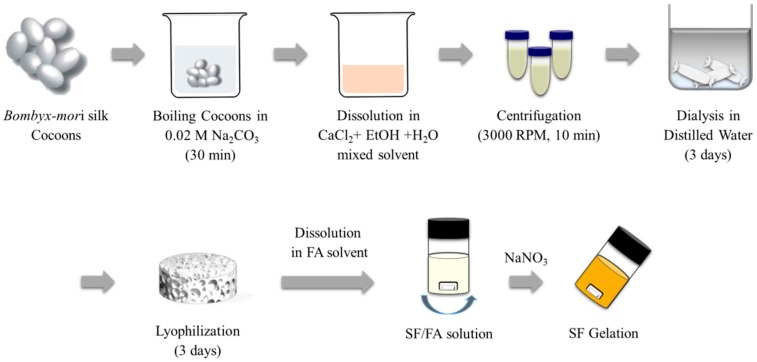
Scheme of SF gelation process using regenerated SF. RPM represents rotation per minute.

Gelation was observed with the transparent SF/formic acid solution that had been prepared with the addition of a variety of salts according to concentrations of 1 wt % to 4 wt %, based on the SF weight (Figure 10). For the effective gelation of the SF, NaNO_3_ was selected as a salt, and its gelation behavior was observed according to the SF and NaNO_3_ concentrations.

### 3.4. Characterization

#### 3.4.1. Viscosity Change Depending on the NaNO_3_ Concentration

The gelation of SF generally induced an abrupt increase in the viscosity. Also, the viscosity of SF gel was associated with the degree of cross-linking. Viscosity changes of the SF/formic acid solution depending on various concentrations (0.1% to 4%) of NaNO_3_ were observed using a viscometer (HADB-III U, Brookfield, MA, USA). The SF concentration was fixed at 5%.

#### 3.4.2. Amino Acid Analysis

SF is consisted of 18 amino acids including Gly, Ala, Ser, and Tyr. Amino acid analysis was conducted to investigate the change in the amino acid composition on gelation. To observe the compositional change, the solvent of SF gel was replaced with water and then analyzed using amino acid analysis (HITACH L-8900, Tokyo, Japan).

#### 3.4.3. UV-Vis Spectrophotometry

Tyrosine and nitrotyrosine residues in the SF gel were able to absorb UV-vis light [31]. On the SF gelation, the absorbance was varied with the tyrosine content. While the NaNO_3_ salt was added to the SF solution, the absorbance change was characterized by the UV-vis spectrophotometer (UV-2450, Shimadzu, Kyoto, Japan). A quartz cell was used and the solution was analyzed at wavelengths of 200 nm to 800 nm.

#### 3.4.4. Fluorescence Spectroscopy

Furthermore, the SF gel was observed using a fluorescent spectrophotometer. To analyze the changes in the structure and composition of the SF gels in both the solution and the dry state, respectively, a fluorescence photometer (Varian cary clipse, Varian, Middelburg, The Netherlands) was used. The excitation wavelength was 365 nm, and the emission wavelength was from 376 to 700 nm.

## 4. Conclusions

The fast gelation of a *Bombyx mori* SF/formic acid solution was induced by the addition of nitrate salts. The salts with nitrate stimulated the consumption of tyrosine and the generation of nitrotyrosine and dityrosine, and this reaction in the tyrosine residue led to an SF organogel. The gelation of the SF was greatly influenced by the amount of NaNO_3_. The SF gelation occurred within a few minutes at below 4% NaNO_3_ in a 5% SF/formic acid solution, but the SF gelation did not occur at above that condition, owing to the viscosity reduction of the gel. In this study, a unique way to promptly and efficiently fabricate the SF organogel is suggested, and if the SF organogel can be transformed to the SF hydrogel by a solvent exchange, the SF gel will be applicable to a variety of fields.
